# Bridging the gap: Using CHNRI to align migration health research priorities in India with local expertise and global perspectives

**DOI:** 10.7189/jogh.13.04148

**Published:** 2023-11-09

**Authors:** Anuj Kapilashrami, Ekatha Ann John, Roomi Aziz, Kit Chan, Kolitha Wickramage, National Advisory Group for MiHSA Priority Setting Initiative, Bontha Babu, Sivakami Muthusamy, Ramila Bisht, Lakshmi Lingam, Irudaya Rajan, Pratap Sharan, Kusuma Yadlapalli, Nima Asgari, Kit Chan, Anns Issac, Anuj Kapilashrami, Kolitha Wickramage, Abha Rao, Ajit Kumar, Amarendra Mohapatra, Ananya Chakraborty, Anindita Chakrabarty, Ankuran Datta, Anna Salomi Kerketta, Archana Roy, Asima Tripathy, Beena Thomas, Bernard D'Sami, Bikash Das, Bontha Babu, D Prabhakaran, Debasish Chowdhury, Divya Ravindranath, Francis Adikalam, Ilango Ponnuswami, Indrani Gupta, Krithiga Shridhar, Lakshmi Lingam, Lekha Subaiya, Leyanna Susan George, Manas Ranjan Behera, Meerambika Mahapatro, Monica Aggarwal, Nilesh Gawde, Paramita Sengupta, Prathap Sharan, Rajeev Sadanandan, Ranjit Kumar Dehury, Ravi Verma, Rekha Chakravarthi, Richa Kundu, Ruma Ghoosh, S Sundari, S Irudaya Rajan, Sabina Singh, Sachidananda Mohanty, Sandeep Sachdeva, Sandhya Mahapatro, Sanjoy Hazarika, Selvam Jesaiah, Shah Ebrahim, Shruthika Murthy, Siddharth Aggarwal, Sivakami Muthusamy, Soniya Kaushal, Harisingh Gour Vishwavidyalaya, Srikanta Kanungo, Suchismitha Mishra, Sunil Raina, T S Syamala, V Prameela, Vidya Venugopal, Vikas Desai, Vishika Yadav, Yadlapalli Kusuma, International Advisory Group for MiHSA Priority Setting Initiative, India Experts Group for MiHSA Priority Setting Initiative

**Affiliations:** 1 School of Health & Social Care, University of Essex, UK; 2 Migration Health South Asia Network (MiHSA); 3 Centre for Global Health, Usher Institute, University of Edinburgh, UK; 4 Migration Health Division, The United Nations Migration Agency (IOM), Switzerland

## Abstract

**Background:**

Migration and health are increasingly recognised as a global public health priority, but concerns have been raised on the skewed nature of current research and the potential disconnect between health needs and policy and governance responses. The Migration Health South Asia (MiHSA) network led the first systematic research priority-setting exercise for India, aligned with the global call to develop a clearly defined migration health research agenda that will inform research investments and guide migrant-responsive policies by the year 2030.

**Methods:**

We adapted the Child Health and Nutrition Research Initiative (CHNRI) method for this priority setting exercise for migration health. Guided by advisory groups established at international and country levels, we sought research topics from 51 experts from diverse disciplines and sectors across India. We consolidated 223 responses into 59 research topics across five themes and scored them against five predefined criteria: answerability, effectiveness, feasibility, impact, and effect on equity. We then calculated research priority scores (RPS) and average expert agreement (AEA) each research topic and theme.

**Results:**

A third of the 59 research topics were on migrants’ health and health care access, 12 on social determinants of migrants’ health, 10 on policies, law and migration health governance, eight on health systems’ responsiveness, and five on migration health discourse. Three of the top five priority topics pertained to migrants’ health care access. The policies, law, and governance theme had the highest overall RPS score.

**Conclusions:**

There is a noticeable gap between research priorities identified by experts at the country-level and the current research focus and priorities set globally. This disconnect between the global and local perspectives in migration health scholarship hinders the development of context-specific and suitable policy agendas for improving migrants' health. Our co-developed agenda emphasises the need to prioritise research on the capacity of existing systems and policies so as to make them more migration-aware and responsive to migrants’ health needs.

Migration and health has emerged as an important global research and policy priority in recent years and is now framed as pivotal to achieving the Sustainable Development Goals (SDGs) 8.8 and 10. Researchers and sectoral experts have called for the inclusion of migrants in global health policies, especially in relation to Universal Health Coverage (UHC) and the broader health and development agenda [1]. The launch of the 2018 Global Compact on Migrants and on Refugees [2,3], the World Health Organizations (WHO) Global Action Plan, and subsequent economic arguments in favor of migrant health policy [4] and migrant-aware systems, policies [5,6] and health services [7] evidences the increased global recognition of migrants’ health needs.

The policy appeals have highlighted the need for a more robust and relevant evidence base and the development of related research capacities [8,9]. These calls also draw attention to the global inequities in evidence around migration and health [2] and emphasise the disconnect between global policies and discourse and the local specificities and realities. For example, scholars have identified critical gaps in knowledge on migration contexts and populations in the Global South in general. For example, regions like Asia have the lowest research output despite having the densest international migration corridors. Almost 80% of the studies within a recent global bibliometric analysis on migration and health came from Global North (USA, UK, Canada, Australia, Germany, Spain, Netherlands, Sweden, Italy, and France), with low-income countries contributing to less than one per cent [10]. Consequently, discourse continues to revolve around migration from low- and middle-income to high-income countries or legality and security-related concerns.

In South Asia, migrants are a highly transient and heterogenous population group, with complex mobility patterns and unique precarious contexts arising from structural inequalities and shared colonial history. These contexts affect migration pathways and health care access and health outcomes in multiple ways [11,12], yet these pathways remain relatively unexplored in research and unaddressed in policies [2]. While South Asia accounted for 15.7% of the global internal displacements in 2021, India had more than 600 million internal migrants and was among the top four countries with most internal displacements [13]. Most of these internal migrants are concentrated in the informal economy, where they engage in low-paying, hazardous jobs, have poor access to water, health care, sanitation, and education, and bear a high burden of discrimination [14,15]. Coronavirus disease 2019 (COVID-19) exacerbated these vulnerabilities, placing a disproportionate burden on mobile populations – while their health remained peripheral in the pandemic responses [16,17], they continued to be pathologised as vectors of disease and framed as villains [18].

Critical gaps in data and health information systems hinder the development and implementation of migration-aware policies and limit any opportunities to drive evidence-informed policymaking. The COVID-19 pandemic highlighted this disconnect, where universal precautionary and relief measures adopted by national governments failed to account for the health and social care needs of mobile populations in resource-poor contexts. Recognising this disconnect in global discourse from regional/local migration specificities and mobility trends, experts have called for the development of and investment into a clearly defined migration and health research agenda to inform migrant-aware policies [2,19-22].

Acknowledgment of these gaps establish the imperative for a local research priority-setting to address the critical policy vacuum at regional and national levels. Against this backdrop, the Migration Health South Asia (MiHSA) network initiated a process to develop regional migration health research agenda through country specific processes, starting with India. It was established in 2019 with the core mandate and vision of building communities of knowledge and practice and capacitating them to address evidence and policy deficits in migration and health at the regional level. Besides building capacities of early and mid-career scholars through workshops, summer schools, and seminars, MiHSA is spearheading initiatives to bring forth evidence on complex patterns of migration in South Asia and its intersection with health, while recognising migrants’ precarity and agency. These include systematic evidence mapping, policy responsiveness assessment and research priority-setting.

We used the Child Health and Nutrition Research Initiative’s (CHNRI) research priority-setting method, a leading systematic and democratic approach in setting health research priorities [23,24], to collate and prioritise research topics to guide investment in related research. The simple and transparent scoring process helps determine points of greatest consensus and disagreement. Originally designed for child health and nutrition research, the method brings together many competing health research ideas with the aim of reducing disease burden and inequities that exist in a population in a feasible and cost-effective way [23]. This is done by engaging a diverse group of stakeholders in the process to bridge the gap between governments, funders, researchers, policymakers, implementers, and those on the receiving end of health research products. Additionally, CHNRI’s conceptual framework is flexible and can be modified for different contexts and multiple health challenges.

Adopting this method provides an opportunity to identify critical, yet neglected areas in migration and health scholarship in India, to embed migrant health more firmly in the national agenda, and strategically align the research agenda with the core preamble of Agenda 2030, of “leaving no one behind”. Here we describe the research priorities in migration and health in India solicited through the CHNRI method to address the regional evidence gap and accelerate translation of this evidence into migrant-aware policies and practices for India by 2030.

## METHODS

**Figure 1:**
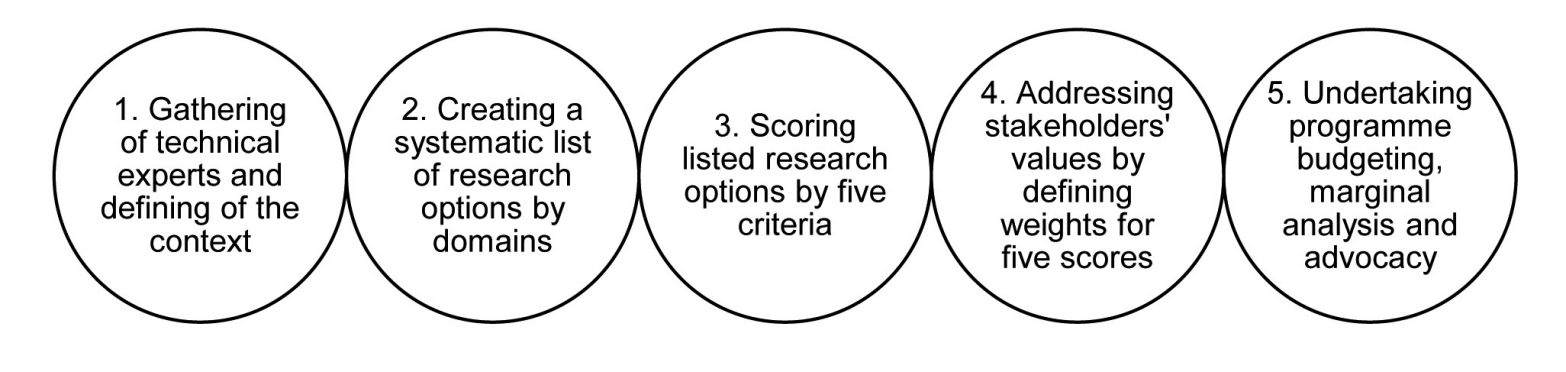
Key Steps of the Child Health and Nutrition Research Initiative exercise followed to identify migration health research priorities for India [26,29].

We adapted this research prioritisation exercise from the CHNRI method as per Rudan et al. [25,26], using Yoshida et al. [27,28] methods of engaging researchers and stakeholders (**Figure 1**).

### Gathering of technical experts and defining of the context

We set up two advisory groups at the outset to guide the CHNRI adaptation process and assist delivery of the exercise; a five-member International Advisory Group made up of senior experts in migration and health, identified through MiHSA’s existing networks and collaborations, and a six-member National Steering Group composed of leading academic and policy experts in the field of migration health in India (Note S1 in the **Online Supplementary Document**).

### Defining the context

**Figure 2:**
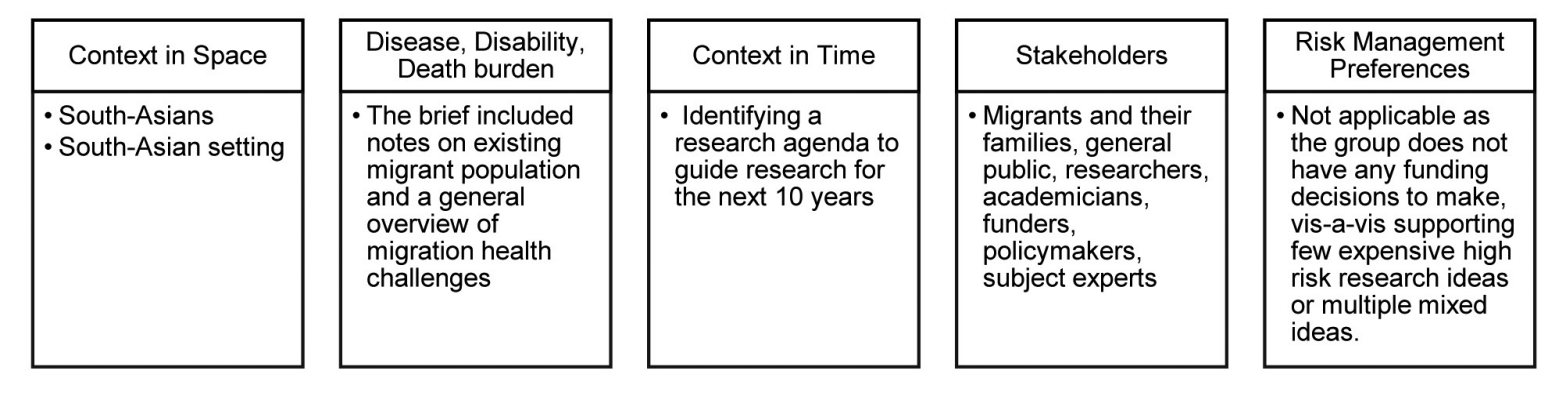
Defining content for setting migration health research priorities.

We developed a technical brief to undertake CHNRI exercise for mapping research priorities for migration and health in South Asia by 2030, identifying the components for context (**Figure 2**).

### Finalising criteria for selecting health research priorities

After setting the context, we sought consensus from the advisory groups on identifying the key criteria appropriate for migration health and South Asia context to rank the research priorities (Note S2n the **Online Supplementary Document**).

### Identifying and inviting subject experts

After defining the context and finalising the criteria, we selected subject experts to list and score research options. We defined “experts” as researchers across disciplines (public health, development studies, epidemiology, social work, anthropology, geography, gender studies, among others) from various institutions in India, academia, civil society representatives working in migration, labor and health spaces, and the government. We used a mix of systematic database searches and snowball methods to identify experts, involving a search of Google Scholar and PubMed using keywords (Note S3 in the **Online Supplementary Document**).

Subsequently, we invited experts to list via an online survey to list three to five distinct research questions/topics that they thought could help India improve migrants’ health and meet related development goals by 2030. The invitation email also offered group authorship for experts who participate in the priority-setting exercise.

### Systematic listing of research options

We solicited 223 research topics from 51 experts, which we then reviewed, excluding nine topics irrelevant to migration and health and discarding duplicates or combining similar topics. This generated a list of 59 priority research topics. We did not differentiate between research questions, research avenues, or research options and labeled them all as research topics.

We then systematically categorised research topics into five research themes through a coding process rather than grouping them according to the original 4D framework usually used in CHNRI process (Description, Delivery, Development, Discovery) [23]. Two research team members independently led the review process and theme allocation, while a third member vetted them, followed by group deliberations around discrepancies and disagreements. Finally, the research topics and themes were shared with the National Steering Group and International Advisory Group for their inputs (Note S4 in the **Online Supplementary Document**):

− Migrants’ health and healthcare access
− Health systems’ responsiveness to migrants’ health needs
− Social determinants of migrants’ health (SDMH)
− Policies, laws & governance of migration health
− Migration health discourse in research and policy

### Scoring of research topics by the five criteria

We invited experts who provided research topics to independently score the 59 consolidated research topics against the set of five pre-agreed criteria (Note S2 in the **Online Supplementary Document**):

1. The topic addresses a significant gap in our knowledge of the field;
2. Evidence generated on this topic can help improve migrants' health;
3. The topic incorporates equity considerations or has the potential to address inequities in processes and/or outcomes;
4. The topic has the potential to directly influence policy, programme/practice and system-wide change;
5. The research is feasible.

### Scoring of listed research priorities by five criteria

#### Calculating research priority scores

We calculated an intermediate score for each research topic by each criterion (five in total) by dividing the sum of the scores (1 = “Agree”, 0 = “Disagree”, and 0.5 = “undecided”) by the number of scorers (excluding those who left their answers blank). We also calculated an overall research priority score (RPS) calculated as the average of the five intermediate scores, converted into percentages (i.e. a range of 0-100), which we then used to rank the research topics.

#### Calculating average expert agreement

**Figure 3:**
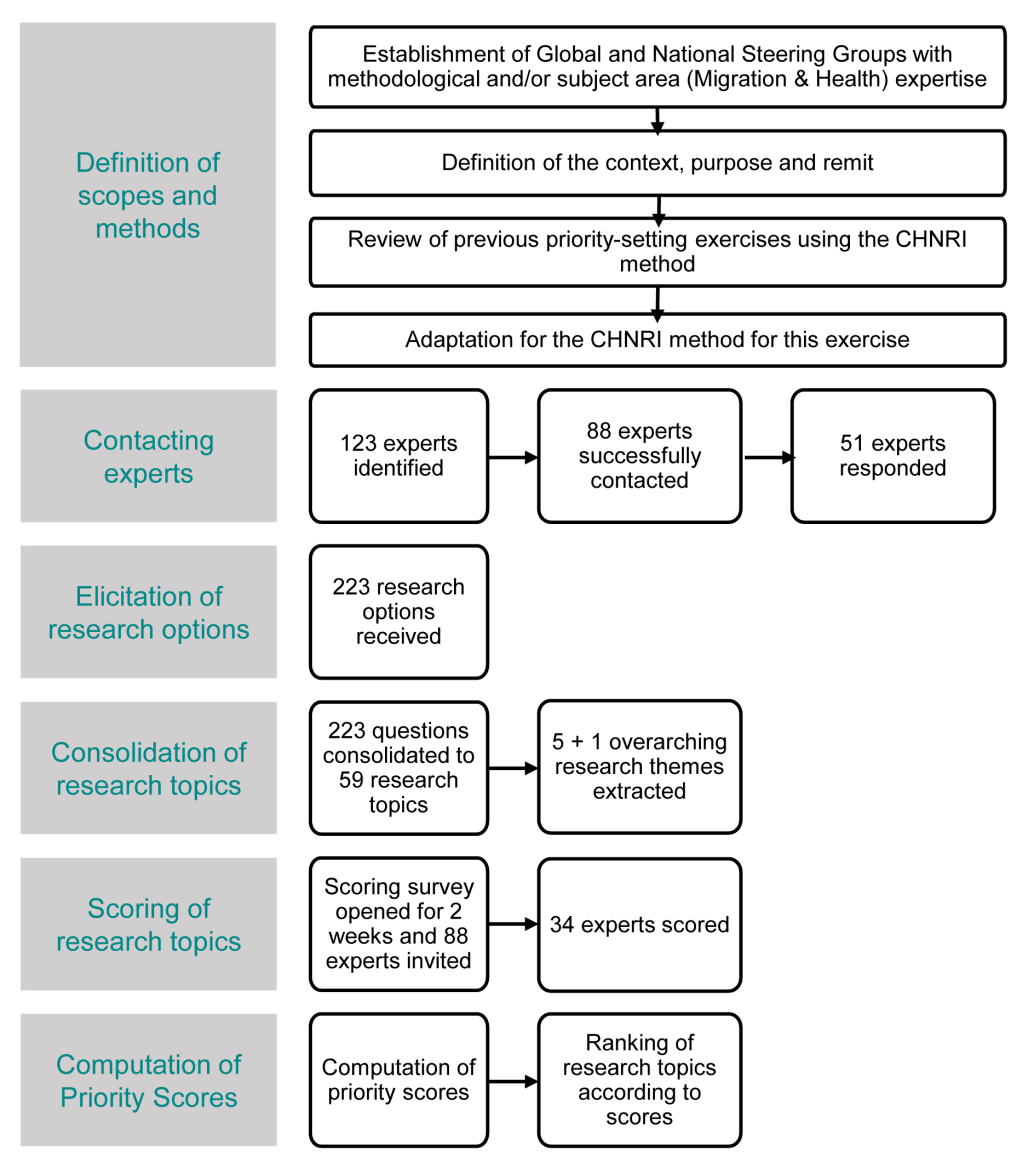
Final process flow followed for the Child Health and Nutrition Research Initiative exercise in India.

We calculated an average expert agreement (AEA) score as a measure of cohesiveness or dispersion in the scorers’ opinion around the most common score (mode):



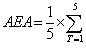



where *T* is the research topic that experts are being asked to evaluate competing research topics. For each evaluated research topic, AEA is informing us what proportion of scorers gave the same most frequent answer for an average (**Figure 3**).

## RESULTS

### Participating researchers and institutions

Of the 51 experts who provided research options, 37 were researchers and 13 were other stakeholders, in total representing 40 institutions (research organisations, academic institutions, government, and non-government organisations). The participants had wide-ranging geographic and disciplinary expertise, representing at least 15 disciplines, mainly community and public health and preventive medicine, developmental studies, epidemiology, demography, social work, and anthropology/medical anthropology.

Of the 34 experts who scored the consolidated research topics in Stage 2 of the exercise, 26 were researchers and eight were representatives of civil society organisations working in the migration, labor, and health space, and the government. As in Stage 1, most of the participants’ had a background in community and public health and preventive medicine, followed by developmental studies, demography, social work, and anthropology/medical anthropology.

### Prioritised research topics

**Figure 4:**
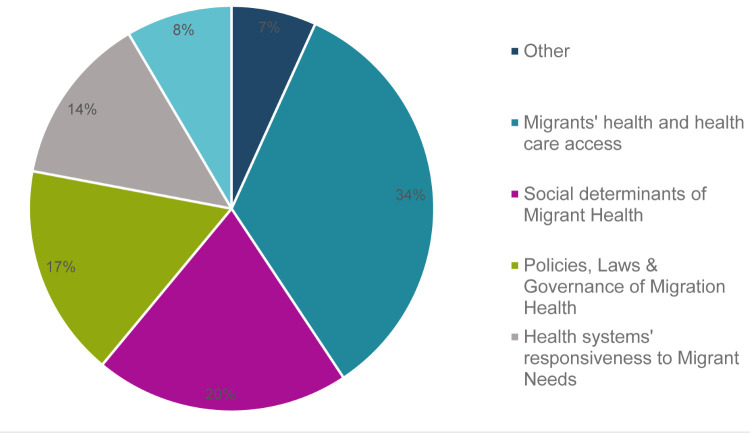
Distribution of 59 research topics by research themes.

As elaborated earlier, we assigned the research topics to one of the five research themes. One-third of the topics (n = 20, 34%) related to migrants’ health and health care access, followed by SDMH [12], policies, law, governance of mental health [10], health systems’ responsiveness to migrants’ needs [8], and migration health discourse in research and policy [5]. Four research topics were marked in the “other” category as they did not fall into the five main themes (**Figure 4**).

### Research priority scores

**Figure 5:**
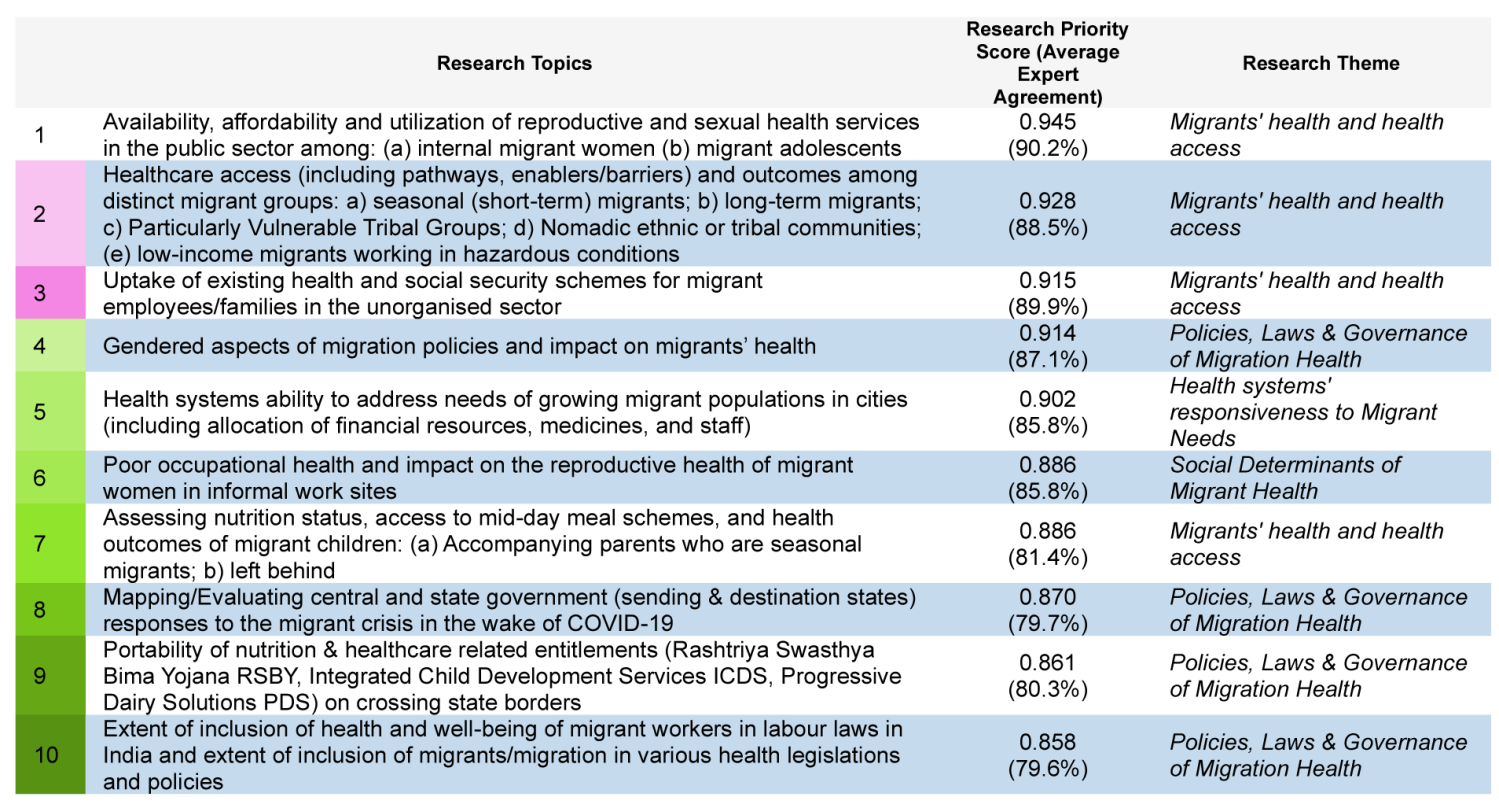
Top ten research topics by research priority scores and average expert agreement.

RPSs for the 59 research topics ranged from 0.945 (highest) to 0.35 (lowest), with a median score of 0.783. Four of the top ten research topics that received the highest overall scores (**Figure 5**) were related to policies, laws, and governance on migration and health, and four to migrants’ health and health care access. One topic from the top ten was on health systems’ responsiveness and one on SDMH (Table S1 in the **Online Supplementary Document**).

**Figure 6:**
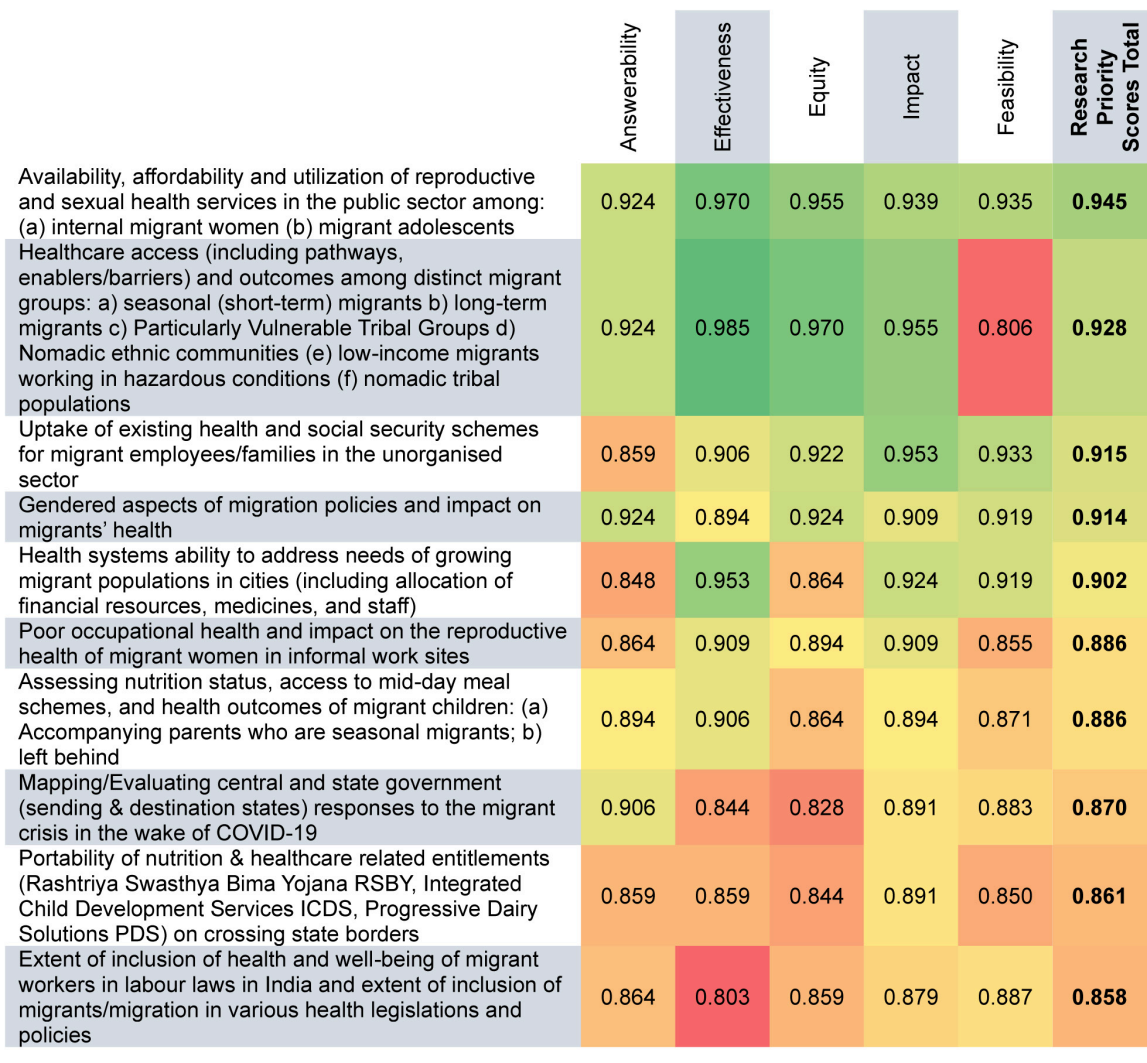
Heatmap of research priority scores of top ten research topics by prioritisation criteria [30].

While the lowest RPS was 0.375, 58 of the 59 research topics scored above 0.5, with RPS scores of maximum topics (n = 42) ranging between 0.729 and 0.915, indicating overall general agreement of experts with the suggested research topics (**Figure 6**).

### Average expert agreement

**Figure 7:**
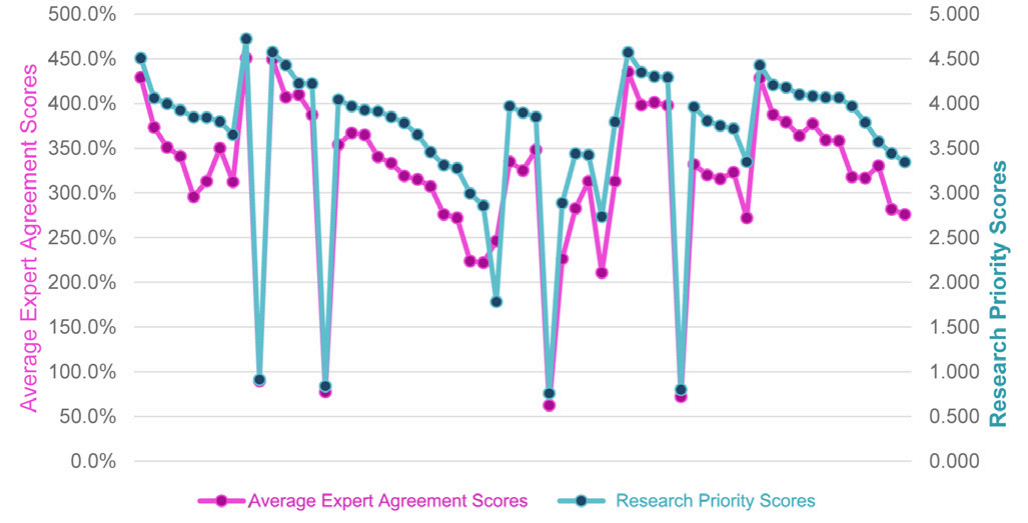
Overlapping average expert agreement and research priority scores of 59 research topics.

The AEA scores ranged from 42.2% to 90.2%, with a median score of 67%, consistent with other CHNRI exercises. Furthermore, the resulting AEA and RPS overlaplped (**Figure 7** and Table S2 in the **Online Supplementary Document**), with a significant and positive correlation of AEA with the RPS (0.912).

**Figure 8:**
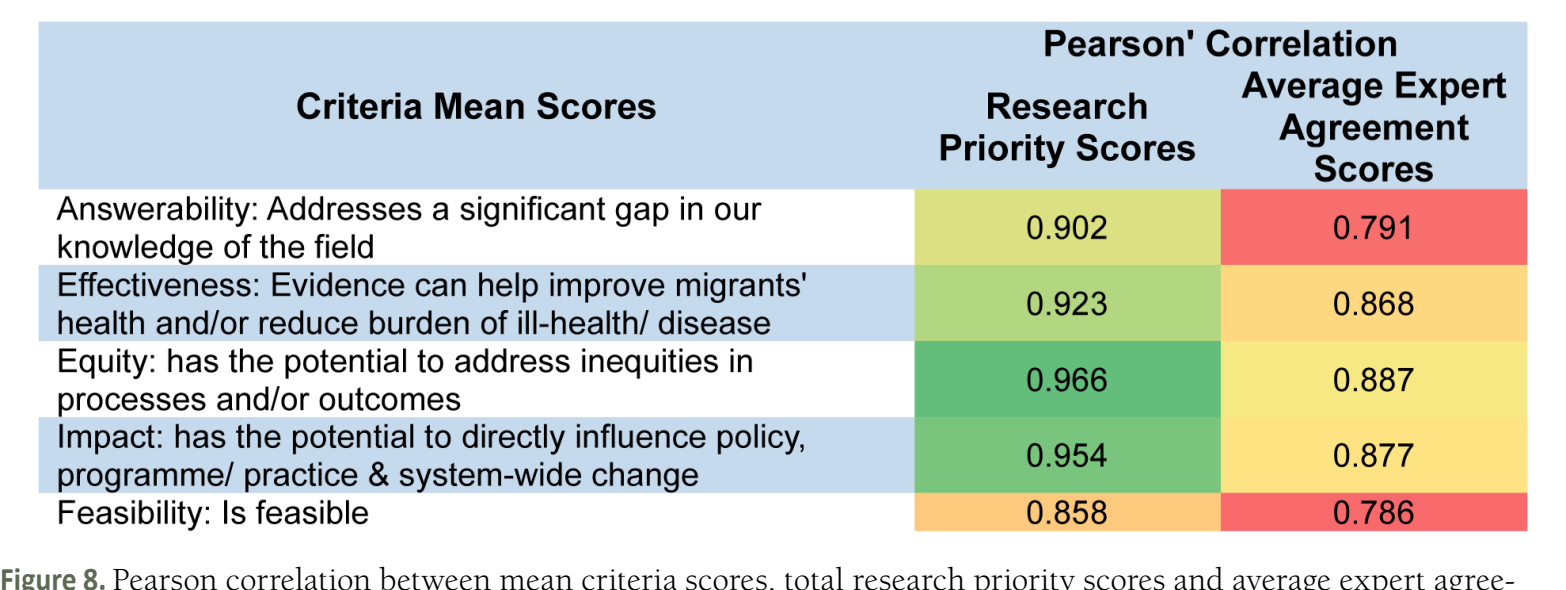
Pearson correlation between mean criteria scores, total research priority scores and average expert agreement scores across the 59 research topics.

We also performed Pearson correlation between RPS, AEA, and mean criteria scores and found overall positive correlation, with the lowest observed between AEA score and answerability dimension of the final research topics and the highest between RPS and potential to address inequities in the processes or outcomes (**Figure 8**).

### Theme and criteria scores

**Figure 9:**
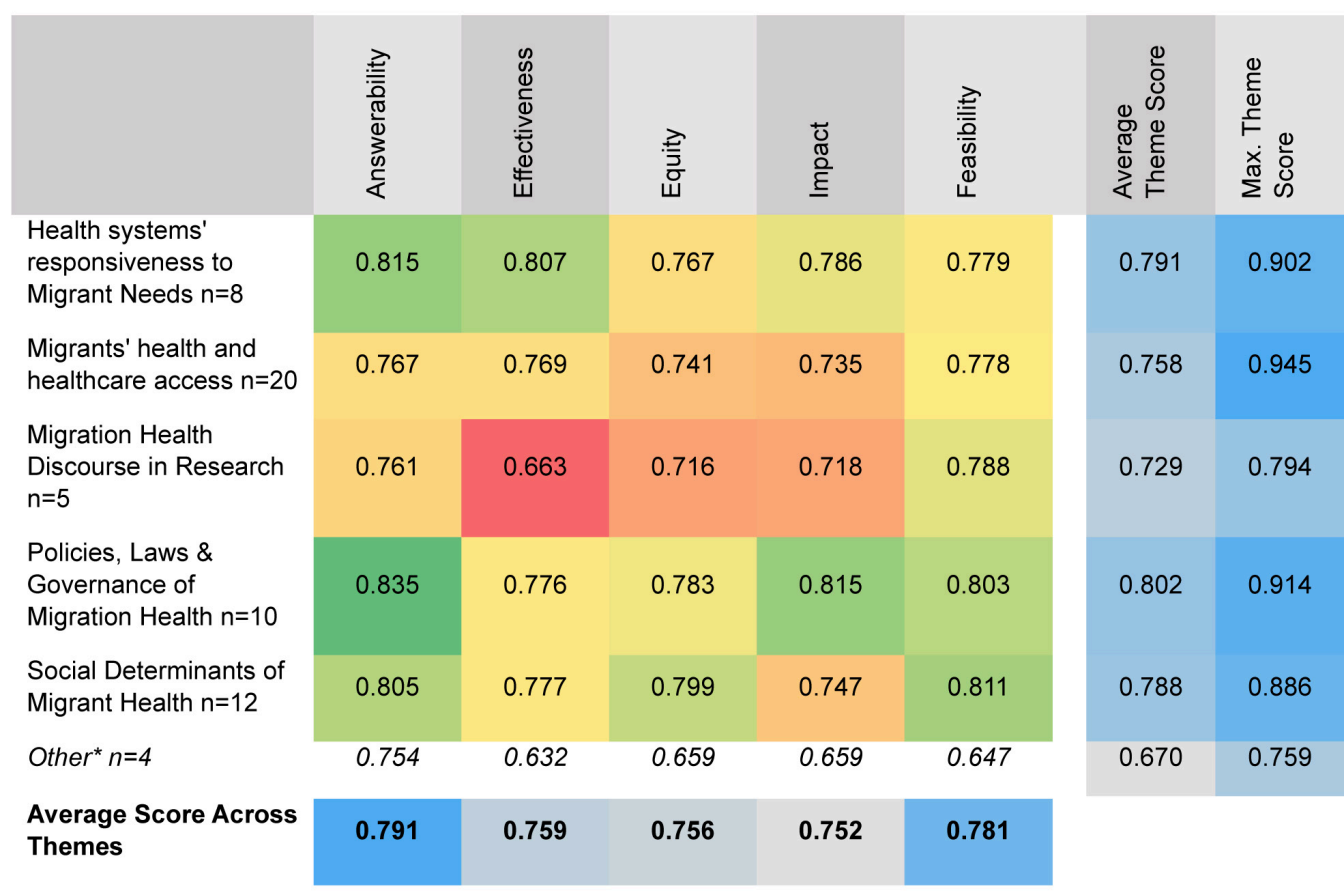
Heatmap of research priority scores between criteria and theme.

We also mapped RPS across the five criteria and the five research themes. While the policies, law, and governance theme received the highest RPS score on average, with experts agreeing to the significant gap in our knowledge that will be addressed through these topics, only one research topic from this theme made it to top 10. The research theme of migration health discourse in research and policy received the lowest RPS score and the lowest score on impact potential, effectiveness, and equity (**Figure 9**).

We did not color-grade the “other” category for visual clarity. We then ranked the research topics by each theme and by the criteria mean scores (Tables S3-S4 in the **Online Supplementary Document**). Overall, the 59 research topics on average scored 0.791 on answerability, 0.781 on feasibility and 0.759 on effectiveness, scoring the lowest on impact (0.752) and equity (0.758). This also reflects on the types of research options proposed in the first place, which might address a specific gap, be effective and feasible, but may not necessarily be incorporating equity considerations or have potential policy impact. Notably, we shared the five scoring criteria with experts only when they were invited to score the research priorities, and not in the first round when they were invited to suggest research options.

### Coverage of migrant categories

Fifteen research topics pertained to internal migrants and six to international migrants, while five topics concerned both. Eighteen topics made the migrant groups of interest explicit, e.g. Bengali migrants in Assam (#50), migrant workers in Gulf (#53, #34), refugees or displaced communities (#21,#41,#47,#29), seasonal migrants (#34), urban migrants (#24,#5,#22,#16), migrants living in slums (#57), and others. These appeared to reflect a focus on groups that may be experiencing heightened precarity attributed to their minority and social status, unfavorable policy/ political environment, poor development infrastructure, and transience. Two of the topics focussed on families of migrants instead of migrants themselves (#32 and #56). Three research topics focussed on adolescents and children, while two outlined different age groups (children, adults and elderly) and 54 did not specify any specific age group.

### Intersecting themes

Gender emerged as a cross-cutting priority across all the themes. Twelve topics drew attention to study of the gendered implication of migration, in alignment with SDG 5.1 and 5.6. In terms of health focus, mental health also emerged as a high priority amidst the experts within 10 research topics. Three research topics addressed both gender and mental health (#18, #24, and #46). Four research topics dealt with infectious diseases (not including COVID-19) (#37, #57, #44, and #34), with two falling in the health systems’ responsiveness theme and two in migrants’ health theme, highlighting the need to study prevalence and pathways of risks/exposure to infection (tuberculosis, helminthic diseases) in migrants, rate of infection, and barriers to surveillance for achieving continuum of care. Despite the prevalent discussions of migrants and climate crisis, only one research topic identified mapping of climate-related migration in India and impact on migrants’ health (#39), under SDMH. Three topics pertained to COVID-19 pandemic (#8, #12, #20), with interest in health systems’ and governments’ responsiveness to migrant crisis and studying mental health challenges in migrants during COVID-19. Only one study specifically focussed on pandemic preparedness (#8), focusing on governments’ strategies to respond to internal migrants during COVID-19, but not highlighting preparedness for future pandemics per se. Eleven of the research topics were addressing UHC, i.e. of ensuring the physical and mental well-being of migrants as their right to health, availability, affordability, and utilisation of health services, the responsiveness of health systems, access and utilisation by migrant populations, health insurance, and others. Seventeen research topics focus on promoting healthier populations, i.e. studying how different determinants affect migrants’ health and their inclusion/exclusion from services and policies (SDMH), and how optimal evidence-informed policy design and implementation can correct this course (Table S1 in the **Online Supplementary Document**).

## DISCUSSION

The research prioritisation exercise for migration and health resulted in a list of 59 relevant research topics provided by national sectoral experts categorised into five themes, with one-third of the topics falling under migrants’ health and health care access, 20% SDMH, 17% under policies, laws, and governance of migration health, 14% under health systems’ responsiveness to migrant needs, and eight percent under migration health discourse in research. Of the top 10 research topics receiving the highest overall RPS, four relate each to migrants’ health, and to policies and governance. All the top three RPS-ranked topics pertain to migrants’ health and health care access, along with high AEA scores. This RPS scoring was done based on answerability, effectiveness, equity, impact, and feasibility of the research topics. Three key lessons can be discerned from our findings, related to methodological innovation in priority setting (including the promise and limitations of CHNRI method), migration health research agenda, and research translation and policy implications of findings.

### Methodological innovation in priority-setting

While CHNRI has been previously used for national-level research priority-setting exercises in India [29-31], these have focussed on its traditional domains of maternal, newborn, and child health, sexual health, mental health, dementia, or infectious diseases, to name a few [32]. Internationally, the Delphi method has been used to identify policy approaches for improvement of migrants’ health [5]. However, consensus development achieved by CHNRI among a larger group of experts using a simple scoring system offer particular advantages. To our knowledge, this is the first time globally that CHNRI is employed to solicit and prioritise research topics for migration health, an emergent but relevant public policy field.

The method helped develop a research priority database that reflects collective optimism on topics of researchers from academic institution, civil societies, and other stakeholders, a failure of current mainstream scholarship and agenda setting [2]. This collective research agenda could help support evidence-informed policymaking at sub-national and national levels.

The research prioritisation exercise involved experts representing several disciplines and regions in India, especially the states with the highest recorded migration rates. However, we observed a clustering of research and policy institutions working on migrants’ issues in big cities in the country’s southern and western regions. Even though snowballing enabled accessing researchers from states not represented in the first round, and we noted that the participants’ research expertise had a wider geographic scope than the city they were located in, leading to the northern-most and northeastern states being under-represented in this exercise. This suggests the need for appropriate research structures at sub-national levels and targeting initiatives to build research capacity in neglected regions. Incentivising participation from under-represented states can help correct the representational balance in migration health research.

Similar to other democratic prioritisation methods, a limitation of CHNRI is its time-consuming nature. Although a systematic crowd-sourced process, ranking 59 topics against five criteria is a time-consuming process and may have impacted the experts’ interest or intent of continuing with the exercise, because of which only 34 out of 51 experts who submitted topics ranked the final topics. Recognising this drawback, we have modified the process to create a digitised form of a Microsoft Excel sheet (Microsoft Corporation, San Francisco, USA) with a drop-down menu for easier ranking for roll out of CHNRI in other countries in the South Asia region.

The exercise raises important questions on the value placed on research topics that are more complex, difficult to answer in the political climate, and consequently less feasible. The top ten research topics indeed sought to answer fundamental questions around availability, accessibility, and utilisation of health services by different migrant populations, factors enabling or hindering their access, and their inclusion in government policies or COVID-19 response schemes. However, questions related to migrants’ participation in social and political processes, macro-environment and processes such as labor regulations and supply chains, funding landscape, or aspects of migration health governance received lower scores. Lower prioritisation of topics that are critical to advancing migration health governance can be attributed to the challenges of doing research in the precarious and transient contexts that migrants inhabit and the impenetrability of the related policy and governance landscape.

### Migration health research landscape

The research priorities identified by regional experts emphasise areas that are often missed in global initiatives for setting research agendas and priorities. For example, the Lancet Migration European Regional Hub’s migration health research priorities for 2020-2021 were UHC and climate change. In contrast, our exercise in India, home to 600 million internal migrants and displaced populations [33], identified only one of the 59 research topics focussed on climate and 11 making a case for UHC, a category deemed critical in the recent WHO evidence on migration and health [34]. Another interesting observation is that despite the timing of this exercise (i.e. during the pandemic), only three topics were related to health emergency preparedness, two of which were specific to responses during COVID-19 (i.e. mapping government responses to migrant crisis during COVID-19 (#8) and migrants’ health and social care needs during COVID-19 (#20)), while the third focused on inclusion of migrants in health policies and legislations (#10), not explicitly in relation to emergency preparedness, suggesting a need for departure from this focus.

We further observed a disconnect between current focus of global and national research and the actual need/priorities for improving migrants’ health. Bibliometric analysis on global evidence on migration and health showed an overwhelming focus on mental health (47% of the 21 457 archived studies) and infectious diseases (13.7%), with issues such as mental health, human immunodeficiency virus/acquired immunodeficiency syndrome (HIV/AIDS), disparities/inequalities, or discrimination dominating in author keywords [10]. A similar exercise undertaken for South Asia region found that migrants’ health continues to be examined through the narrow lens of infectious disease, along with growing focus on mental health, albeit limited to refugees and incarcerated migrants [11]. Such focus on infectious diseases is unsurprising, as migration health continues to be located in health emergencies departments of intergovernmental health agencies in many regions. Our findings challenge the assumed importance of these health conditions. Only four research topics listed infectious disease, none of which made it to the top thirty ranking research topics.

Migration awareness and migrant-responsiveness of existing policies and health systems found renewed traction during COVID-19, where relief measures introduced tended to ignore migrants in precarious situations [16]. While the historic neglect of migrants in public policy on account of lack of official registration by their employers is well established, COVID-19 widened this gap [11,12,35]. This exercise renews attention to safety-nets for intra-, inter-state as well as cross border migrants.

The responses also confront the general tendency in migration health literature to dichotomise migrants vs locals, presenting both groups as homogenous. In the process, policies and research tend to erase migrants’ unique contexts of precarity, structural vulnerabilities and agencies. Explicit references to specific migrant population groups like Bengali migrants in Assam, nomadic ethnic communities like Nats and Gujjars, Gulf-return labor migrants draw attention to various social divisions and structural inequalities defining migrants’ precarity, mobility patterns, and their health in India. Experts recognised the heterogeneity among migrants in India, where they represent diverse caste, ethnicity/indigeneity, and religion. These divisions operate as fault lines in migration governance and disease management and become critical considerations for designing and implementing research. Even across the 12 research topics mainstreaming gender, experts incorporated intersectional perspectives like increased vulnerabilities due to informality of work, socio-economic status, caste, religion, adolescence, and of families left behind. The identification of gender as a key access of studying vulnerability among migrants is in line with the growing attention to gender in labor migration scholarship in India [36]. This exercise affirms the clear need to steer funding into research on the broader context of migration and the structural impediments migrants face while accessing health care [2].

Our findings also established the merit in branching out from broad and generic regional priorities (i.e. collating research priorities under an overarching and “over-simplified” Global South umbrella) to focus on specific and unique country contexts to drive local funding into relevant research addressing structural challenges leading to migrants’ poor health and health care access. MiHSA is undertaking similar country-level CHNRI exercises in the wider South Asia region facing similar challenges across distinct population groups and political contexts – for example, in Pakistan, with the access problems faced by the Afghan refugee crisis and Bengali settlers in the country within the wider political context, or in Nepal, with health care access challenges of seasonal labor migrants traveling to and from India.

### Research translation and implications for migration health policy

As with all research agenda setting tools and processes, defining research priorities/ agenda in itself cannot advance migration health. Advancing an evidence-based agenda for health of migrants needs concerted advocacy with and buy-in of policy makers, funders, and international agencies, and the political will to allocate resources to empirical research on identified priorities and uptake of research evidence for migration aware and migrant-responsive policies and systems.

Such research-evidence-policy translation cycle requires a research governance ecosystem that is embedded in migration health governance and policy domains. Countries in the South and Southeast Asian region (e.g. Sri Lanka, Philippines, Nepal, and Vietnam) offer promising roadmaps for such translation. For instance, the National Migration Health Secretariat and Taskforce (NMHT) in Sri Lanka [37] and the Intra-Agency Task Force on Migrant Health in Philippines (Philippines Migrant Health Network) [38] served to institutionalise an inter-sectoral approach that engaged policymakers, local researchers/institutes, advocacy networks, and migrant communities to inform the policy and programmatic responses. These inter-sectoral, inter-agency and interdisciplinary coordination platforms became conduits to bring together diverse stakeholders within both migration governance and health governance ecosystems to drive research agendas for migration health interventions, action, review and evaluation. For example, NMHT forged a migration health research agenda by establishing a national Migration Health Research Commission. The commission, chaired by the Ministry of Health, engaged government ministries, local and international academics, civil society, migrant organisations, and UN agencies over a three-year process to distil research evidence on health impacts and determinants of inbound, outbound, and internal migrants in Sri Lanka and their families. Research findings distilled were presented at national consultative forums and discussed through technical working groups. The data points informed the national migration policy formulary and action plan. Migrant voices and experiences were also critical, not simply layering empirical data at these fora. For instance, the migrant families research strand of the National Research Agenda revealed how the caregivers of children of migrant workers in precarious employment contexts were predominantly elderly, with over 30% suffering from generalised depression, anxiety, and somatoform disorders promoting policy makers and practitioner to support “who cares for the elderly caregivers” within labor migration process [39,40].

These processes are critical for integrating research ecosystem within the policy domain, ensuring the translational value of research and providing technical policy direction for advancing migration health.

In federal systems such as India, such platforms must be established at both national and sub-national levels as the migration dynamics differ greatly across states and regions. It is also important to ensure that these mechanisms are not re-imagined as vectors of top-down agenda setting where policy makers dictate the research agenda. Appropriate structures to facilitate meaningful engagement of migrant populations is necessary in driving this agenda. The formation of meaningful “bottom up and inside out” networks described above requires both political and academic will, and willingness of policy makers and researchers to collaborate on driving evidence-based migration health research agenda [39]. Such networks can also serve as powerful monitoring mechanisms for migration health action.

## CONCLUSIONS

The production and use of evidence on migration is steeped in global inequities [2]. Research funding, agenda-setting, and practice disproportionately favor institutions in high-income countries [10]. Within South and South-East Asia, migration health continues to be a relatively under-explored topic, with limited attention given to the health and social care needs of the highly transient and heterogeneous migrant population, or to the geographical and contextual specificities of the region. Starting with India, we used this research priority-setting exercise to identify critical evidence needs to effectively inform policymakers and funding organisations on relevant policies and programs on migration and health. In contrast to the migration health discourses in the “global North” that emphasise communicable diseases and mental health of primarily international migrants, Indian researchers place emphasis on policy, programmatic support, infrastructure and the recognition of rights and entitlements to surmount access issues. While this exercise has yielded a systematically co-developed national research agenda for India, it also warrants further steps, including increased investment, infrastructure development, capacity building, and international (South-South and South-North) partnerships for successful knowledge development. Nevertheless, our findings can inform funding decisions for evidence generation and lay the foundations for utilisation of evidence for migration-aware and migrant-responsive health and social policies.

## Additional material


Online Supplementary Document
