# Supplementary material for: Bridging the gap: Using CHNRI to align migration health research priorities in India with local expertise and global perspectives
Source: J Glob Health. 2023 Nov 9;13:04148. doi: 10.7189/jogh.13.04148 (PMC10630695; doi:10.7189/jogh.13.04148)
Supplement: Online Supplementary Document [file jogh-13-04148-s001.pdf]

ONLINE SUPPLEMENTARY DOCUMENT

Note S1 – Research Steering Group

Constitution of International Steering Group

Members

1. Dr Nima Asgari - Director, Asia Pacific Observatory on Health Systems & Policies (APO), World Health Organization (WHO) South-East Asia

2. Dr Kolitha Wickramage - Global Migration Health Research & Epidemiology Coordinator, UN- International Organisation for Migration

3. Dr Anns Issac - Technical Officer, APO & WHO Regional Officer for South-East Asia

4. Dr Kit Yee Chan - Reader, University of Edinburgh

5. Prof Anuj Kapilashrami - MiHSA (Chair), Professor, Global Health Policy and Health Equity, University of Essex

National Steering Group

1. Dr Bontha V Babu - Scientist-G and Head of Socio-Behavioral & Health Systems Research (SBHSR) Division, Indian Council of Medical Research (ICMR)

2. Prof Y S Kusuma - Centre for Community Medicine, All India Institute of Medical Sciences (AIIMS)

3. Prof Pratap Sharan - Department of Psychiatry, AIIMS

4. Prof Lakshmi Lingam - Tata Institute of Social Sciences (TISS)

6. Prof S Irudaya Rajan - Chairman, International Institute of Migration and Development

7. Prof M Sivakami - School of Health Systems Studies, TISS

Activities undertaken with the Steering Groups

The International Advisory Group was met with to finalize the overall MiHSA plans for undertaking CHNRI exercise in the region, review the criteria for scoring of priorities.

The National Steering Group was met with thrice, first to review and finalize the criteria for scoring of priorities and advise on expert identification strategy, second time to review and share feedback on the research themes, and third time to review the ranking. Phase-wise written reports were shared with the National Steering Group for their feedback and comments.

Note S2 CHNRI Ranking Guidance Note for the Five Pre-Defined Criteria

Following the standard CHNRI method, five scoring criteria were selected and agreed upon for this exercise. While the CHNRI method recommends five criteria as a standard (answerability, effectiveness, deliverability, potential impact and equity (Rudan, 2016), for the purpose of this exercise we replaced deliverability with feasibility. Considering the paucity of data around migrants' health, difficult access to specific migrant groups and risks associated with exposing migrants in precarious settings to additional vulnerabilities during research, we identified feasibility as a criteria to rank topics that can be researched, practically and ethically i.e. accessibility to study participants, availability of data etc. while continuing with five criteria in total. Feasibility was found to be the most frequent deviation in original CHNRI recommendations (Rudan *et al.*, 2017). Furthermore, the potential impact on disease burden was modified to potential policy impact (See Appendix IV for CHNRI ranking guidance shared with experts).

The scoring options available were as follows: 1 for "Agree," 0 for "Disagree," 0.5 for "Undecided," and leaving the field blank indicated a lack of expertise to score

The topic addresses a significant gap in our knowledge of the field:

Migration health continues to be a relatively underexplored area of research in South Asia (*Kapilashrami et al 2020*), with limited attention given to the health and social care needs of migrants – who are a highly transient, diverse and heterogeneous population group. While migration health scholarship in India is growing, it's still in its nascence and marked by critical gaps. Some health issues, and migrant groups tend to be a greater focus, while other critical issues remain unexplored or under-researched. Certain disciplines and perspectives are also overlooked in the current body of research but remain critical for the advancement of migration health field (*Samaddhar 2020*). This criterion therefore demands a consideration of what is missing in current scholarship, and steers agenda towards addressing these knowledge gaps.

Improvements in health - Evidence generated on this topic can help improve migrants' health and/or reduce burden of ill-health/ disease:

The overall purpose of this exercise is to facilitate research that can help improve migrants' health by enabling more responsive healthcare systems as well as tackling the wider social, economic and political determinants of health. This criterion therefore accounts for the extent to which the research topic will generate evidence, insights and learnings that will can enable such improvements.

Equity implications- The topic incorporates equity considerations or has the potential to address inequities in processes and/or outcomes:

Research has shown that insufficient attention has been given to studying the role of structural inequalities in determining poor access to health care and vulnerability to ill health among migrant groups like internal migrants in India (*Kusuma and Babu 2018*). Studies on migration have often tended to focus on specific migrant groups (international vs internal; economic migrants vs IDPs), ignoring the heterogeneity within and the intersecting aspects of social position (e.g. gender, class, caste, religion, age, indigeneity, sexuality) that shape experiences (*Kapilashrami and Hankivsky 2018*). This criterion

encourages you to examine whether the topic goes beyond the scope of narrow public health intervention, and allows capturing the diversities and the complexities of migration, as well as migrants' precarity, vulnerabilities and agency.

The topic has the potential to directly influence policy, programme/ practice and system-wide change:

There's currently a stark disconnect between the production of knowledge by researchers and its use by policy-makers (*Kapilashrami et al 2020*). This disconnect and dearth of information can hamper effective policy and programme development and may even undermine the impact of any actions taken. The convergence of migration policy with other areas such as health and labour at global level has created the need for policy-makers to engage in cross-sector dialogue to align priorities and coordinate responses. This criterion examines whether the topic provides a crucial evidence base that can inform inclusive and effective policies and institutional responses, and has the potential to identify and address gaps in a broad spectrum of service delivery and the extent to which it's aligned with questions being asked by policy-makers and practitioners (across a wide spectrum of government, civil society institutions and funding bodies).

The research is feasible:

This criterion looks at the overall feasibility of this research. The topic is doable in terms of accessibility to participants and availability of data, and the area of enquiry does not increase migrants' vulnerabilities or pose harm to them.

Note S3 - Experts' Identification Strategy

We used a mix of systematic database searches and snowball methods to identify experts, particularly from academia and research. The former method involved a search of **Google Scholar and PubMed,** using variations of the keywords "health", "wellness" and "well-being" with "India/ Indian" along with variations of the term migrants, including "internal migrants" OR "migrant workers" OR "refugee" OR "stateless people" OR "international student*" (see below).

Example of keywords used:

Keyword Category	Keywords
Health	Health* OR Well?Ness OR Well?Being OR disease
AND	
Context	India*
AND	
Migrants	Migr* or Emigr* Or Immigr* OR refuge* or State?Less

Search was done on Google Scholar and Pubmed.

Relevant articles were reviewed and details of their first, second and third authors were extracted. From the archived authors, those with at least three publications, either as first, second or senior author) in peer–reviewed journals from 2010-2021 were selected. The search was continued until no new authors were coming up. This search was supplemented by manual extraction of publicly available reports on migration and refugee health in India (e.g. from government websites) to identify experts from civil society organizations and the government. In addition, members of the National Steering Group provided names of scholars from regions and institutions under-represented in policy planning to be added to the list of experts. Based on these approaches, an initial list of 95 experts was developed.

As we started reaching out to these experts, we also asked them to nominate names of other relevant experts/stakeholders, especially those from civil society organisations that work with refugees and low-income workers in the informal economy, and the government sector, who may have been inadvertently excluded in our search, yielding an additional 28 names.

The final experts were plotted into an excel spreadsheet with details of their disciplines/ specialties, publication, institutional affiliation and contact details.

Note S4 – Describing the Research Themes

Theme 1 – Migrants' health needs and healthcare access

This theme includes topics related to the specific health issues experienced by migrants including prevalence and burden of chronic and infectious diseases, health care access, barriers to utilization of health services, and outcomes among distinct migrant groups in India.

Theme 2 – Health systems' responsiveness to migrant needs

Topics included in this theme probe the ability of health systems to address the complex needs of migrant populations, including the allocation of financial resources, medicines, and staff. The theme also explores health system response to migrants during the COVID-19 crisis. These topics can shed light on the migration awareness and responsiveness of health systems to the needs of migrant populations and the challenges faced in achieving equitable health outcomes.

Theme 3 – Policies, laws and governance of migration health

Topics included in this theme exact examination of the legal and policy frameworks that govern the health of migrants, including immigration policies, health policies, and social welfare policies. They cover both design and implementation/ enforcement of these policies, governance structures as well as evaluations of their effectiveness. For the purpose of consolidation, topics dealing with the legal and ethical frameworks governing the rights and protections afforded to migrants, including their right to health care and other social services were also included under this theme.

Theme 4 – Social determinants of migration health

Topics included in this theme address specific social locations of migrants as well as wider political, commercial, environmental, economic factors when migration itself is interacting with these factors and determining health, such as their socioeconomic status, living and working conditions, cultural and language barriers, and exposure to discrimination and violence. This topic also explores how societal and political structures/factors impact the health outcomes of migrants, including discrimination, stigma, and access to social services.

Theme 5 – Migration health discourse in research and policy

Topics that examine how migration health is conceptualized and how migration (and migrants) are framed in public and policy discourses as well as in research. This theme encompasses challenges and opportunities in developing a research agenda on migration and health, the discursive framing of the intersection of migration and health governance and how this influences policy responses.

Table S1- List of Final Topics, Ranking, Scores, AEA and Themes

Rank	Research Topics	RPS	AEA	Research Themes
1)	Availability, affordability and utilization of reproductive (incl maternal) and sexual health services in the public sector among: (a) internal migrant women (b) migrant adolescents	0.945	90.2%	Migrants' health and health access
2)	Healthcare access (including pathways, enablers/barriers) and outcomes among distinct migrant groups:<br>a) seasonal (short-term) migrants<br>b) long-term migrants<br>c) Particularly Vulnerable Tribal Groups<br>d) Nomadic ethnic communities (eg: Nats and Gujjars)<br>(e) low-income migrants working in hazardous conditions<br>(f) nomadic tribal populations	0.928	88.5%	Migrants' health and health access
3)	Uptake of existing health and social security schemes for migrant employees/families in the unorganised sector	0.915	89.9%	Migrants' health and health access
4)	Gendered aspects of migration policies and impact on migrants' health	0.914	87.1%	Policies, Laws & Governance of Migration Health
5)	Health systems ability to address needs of growing migrant populations in cities (including allocation of financial resources, medicines and staff)	0.902	85.8%	Health systems' responsiveness to Migrant Needs
6)	Poor occupational health and impact on the reproductive health of migrant women in informal work sites	0.886	85.8%	Social Determinants of Migrant Health
7)	Assessing nutrition status, access to mid-day meal schemes, and health outcomes of migrant children: (a) Accompanying parents who are seasonal migrants; b) left behind	0.886	81.4%	Migrants' health and health access
8)	Mapping/Evaluating central and state government (sending & destination states) responses to the migrant crisis in the wake of COVID 19	0.870	79.7%	Policies, Laws & Governance of Migration Health
9)	Portability of nutrition & healthcare related entitlements (RSBY, ICDS, PDS) on crossing state borders	0.861	80.3%	Policies, Laws & Governance of Migration Health

Rank	Research Topics	RPS	AEA	Research Themes
10)	Extent of inclusion of health and well-being of migrant workers in labour laws in India and extent of inclusion of migrants/migration in various health legislations and policies	0.858	79.6%	Policies, Laws & Governance of Migration Health
11)	Effect of state-funded health insurance programmes in reducing inequalities (incl Ayushman Bharat) in healthcare access for migrants	0.846	82.0%	Migrants' health and health access
12)	Depression, anxiety, and stress and socio-demographic correlates among internal migrants during COVID-19	0.845	77.5%	Migrants' health and health access
13)	Gender differences in the prevalence of chronic disease risk factors and outcomes based on migration status (type and duration)	0.842	77.6%	Social Determinants of Migrant Health
14)	Work and living conditions (incl sanitation) among low-income migrants and the resulting impact on their health (occupational health)	0.836	75.9%	Social Determinants of Migrant Health
15)	Comparative study of health-seeking behavior, healthcare utilisation and treatment outcomes among: migrants and non-migrants, migrants and other marginalised groups in a particular area, Internal and cross-border woman migrants, recent migrants and long-term migrants, male and female migrants	0.831	71.5%	Migrants' health and health access
16)	Examination of (and tools to assess) the safety and vulnerability of urban migrant families	0.820	72.8%	Social Determinants of Migrant Health
17)	Gender differences in migratory patterns and associated health/social risks	0.817	75.5%	Social Determinants of Migrant Health
18)	Gender-based inequalities (pay, work conditions) among migrants in services sectors and its impact on mental health and well-being of women	0.814	71.9%	Social Determinants of Migrant Health
19)	Impact of intersection of different positionalities (for e.g, socio-economic status, caste, gender, religion) on access to/experience of healthcare	0.813	71.7%	Social Determinants of Migrant Health
20)	Health systems' response to internal migrants during COVID-19 crisis	0.813	74.7%	Health systems' responsiveness to Migrant Needs
21)	Assessment of social & healthcare needs of migrants/immigrants/refugees in a given context of migration (internal labour/cross-border migration/forced displacement due to disasters/conflicts/developmental activities/political evacuations, etc.)	0.809	70.8%	Migrants' health and health access
22)	Rights of adolescent girls of migrant families in urban areas in relation to the United Nations Convention on the Rights of the Child's (UNCRC) framework of rights to survival, development, protection and participation	0.802	72.3%	Policies, Laws & Governance of Migration Health
23)	Mental health burden and health systems response to (a) single male migrants in India's metros (b) seasonal migrants and their children (c) elderly migrants in urban areas (d) migrants engaged in unsafe workspaces	0.800	70.2%	Health systems' responsiveness to Migrant Needs

Rank	Research Topics	RPS	AEA	Research Themes
24)	Factors affecting mental health of single migrant women working in urban areas in both formal and informal economy (incl role of gender)	0.794	63.6%	Social Determinants of Migrant Health
25)	Mapping mental healthcare pathways among migrant communities – barriers and facilitators.	0.794	73.5%	Migrants' health and health access
26)	The conceptual and theoretical underpinnings of research on migration and health: Exploring questions around how ‘migration’ and ‘migrants’ have been defined in academic and policy worlds, and how this relates to on the ground	0.794	67.0%	Migration Health Discourse in Research
27)	Review of global best practices that facilitate portability of sexual and reproductive rights of internal migrants from the place of origin to place of destination	0.792	66.4%	Policies, Laws & Governance of Migration Health
28)	Healthcare expenditure of a migrant household in city and impact of out-of- pocket medical expenses	0.786	73.0%	Migrants' health and health access
29)	Assessing the efficacy/accessibility of health promotion interventions and materials and their uptake by migrants (cross border migrants/ refugees, IDP) in destination states.	0.785	68.2%	Health systems' responsiveness to Migrant Needs
30)	Mapping family planning needs and adverse birth outcomes in migrant households	0.783	68.1%	Migrants' health and health access
31)	Challenges (and opportunities) in developing a research agenda on migration and health in India and influencing policy	0.780	65.0%	Migration Health Discourse in Research
32)	Determinants of decision-making in health among families (especially women, children and the elderly) left behind for migration: a. Within country (seasonal and/or long-term) b. Across borders	0.770	66.7%	Migrants' health and health access
33)	Developing robust evidence base to understand the intersection of migration and health in the absence of datasets	0.770	69.7%	Migration Health Discourse in Research
34)	Barriers to surveillance and effective measures to ensure continuum of care (and address the challenges of treatment initiation and drug adherence) for informal sector/ short-term seasonal migrants suffering from infectious diseases (TB etc)	0.769	59.2%	Health systems' responsiveness to Migrant Needs
35)	Violation of migrant workers’ rights as they undergo mandatory medical tests, including pregnancy tests, in destination countries (particularly Gulf and South East Asian countries)	0.769	62.6%	Health systems' responsiveness to Migrant Needs
36)	Mapping best practices that facilitate cooperation between governments in source and destination state/countries and among government, researchers and policy makers for mitigating health issues of low-income migrants in India	0.762	64.0%	Policies, Laws & Governance of Migration Health

Rank	Research Topics	RPS	AEA	Research Themes
37)	Tracking transmission of diseases such as Tuberculosis during migration	0.760	70.0%	Health systems' responsiveness to Migrant Needs
38)	Mapping demand-supply and experiences of female migrant carers of India's increasing "greying" population	0.759	62.6%	WHAT
39)	Mapping climate- related migration in India and its impact on the health and well-being of communities	0.758	63.4%	Social Determinants of Migrant Health
40)	Difference in nutrition intake and food security at destination and source states/countries and impact on health	0.757	63.9%	Migrants' health and health access
41)	Review of policies & best practices in rehabilitation of refugees, accommodation and inclusion of migrants and policies & practices that hinder these	0.751	63.2%	Policies, Laws & Governance of Migration Health
42)	Governance and funding landscape of Migration and Health in India	0.744	64.6%	Policies, Laws & Governance of Migration Health
43)	The role of digital technology in addressing health disparities among migrants with regard to access to information and care	0.731	62.5%	Health systems' responsiveness to Migrant Needs
44)	Migrants are often blamed for outbreaks by governments. A study on the epidemiology and burden of health conditions, and infection rate among migrants against general population.	0.731	63.0%	Migrants' health and health access
45)	Values, principles and approaches that should guide research agenda on migration and health in India (i.e. human rights, labour rights or public health approach as well as drawing from a range of disciplines including sociology, industrial relations, political science, law, anthropology, psychology etc.)	0.723	60.4%	Migration Health Discourse in Research
46)	Pathways through which migration affects mental health and the intersection of caste and gender	0.715	66.1%	Social Determinants of Migrant Health
47)	Experience of trauma and prevalence of Post-Traumatic Stress Disorder (PTSD) among sections of migrants, refugees and displaced communities	0.692	61.5%	Migrants' health and health access
48)	Migrants' participation in social, political and democratic processes in their destination states/countries	0.689	56.4%	Social Determinants of Migrant Health
49)	Change in health vulnerability and resiliency across the four phases of migration cycle (predeparture, during transit, at destination and upon come to their original place)	0.688	56.6%	Other
50)	Impact of the National Register of Citizens on the mental health and well-being of Bangladeshi immigrants/Bengali migrants in Assam	0.685	62.6%	Other

Rank	Research Topics	RPS	AEA	Research Themes
51)	Mapping the everyday stressors, including struggle for placemaking, effort to ensure better opportunities and identity negotiation that impact the mental health of migrants at the destination	0.670	54.5%	Policies, Laws & Governance of Migration Health
52)	Cumulative impact of different social determinants of health at different stages of migration	0.670	55.2%	Social Determinants of Migrant Health
53)	Health assessment of return migrants from the Gulf (and beyond) and continuity of care	0.663	55.3%	Migrants' health and health access
54)	The role and use of traditional/alternate medicine in increasing access to/utilization of healthcare among migrants	0.655	54.5%	Migrants' health and health access
55)	Mobility, Mortality and pre-disposition to risk among migrant men	0.599	44.8%	Migrants' health and health access
56)	The psycho-social-economic impact of death of migrant workers on their families	0.578	45.3%	Migration Health Discourse in Research
57)	Prevalence & determinants of soil-transmitted Helminthic disease among migrant slum population	0.572	44.4%	Migrants' health and health access
58)	Examining the contribution of migration to demographic transition	0.547	42.2%	Other
59)	Evaluation of the serum cytokine profile of migrants of endemic and non-endemic areas and changes in immunity	0.357	49.3%	Migrants' health and health access

Table S2 – RPS and AEA scores of Research Topics across Research Themes

Research Topic Scores			Research Themes					
Topic IDs	RPS	AEA	Health systems' responsiveness	Migrants' health and health access	Migration Health Discourse in Research	Policies, Laws & Governance of Migration Health	Social Determinants of Migrant Health	Others
1	0.945	90%		1				
2	0.928	89%		1				
3	0.915	90%		1				
4	0.914	87%				1		
5	0.902	86%	1					
6	0.886	86%					1	
7	0.886	81%		1				
8	0.870	80%				1		
9	0.861	80%				1		
10	0.858	80%				1		
11	0.846	82%		1				
12	0.845	78%		1				
13	0.842	78%					1	
14	0.836	76%					1	
15	0.831	71%		1				
16	0.820	73%					1	
17	0.817	76%					1	
18	0.814	72%					1	
19	0.813	72%					1	
20	0.813	75%	1					
21	0.809	71%		1				
22	0.802	72%				1		
23	0.800	70%	1					
24	0.794	64%					1	

25	0.794	73%		1				
26	0.794	67%			1			
27	0.792	66%				1		
28	0.786	73%		1				
29	0.785	68%	1					
30	0.783	68%		1				
31	0.780	65%			1			
32	0.770	67%		1				
33	0.770	70%			1			
34	0.769	59%	1					
35	0.769	63%	1					
36	0.762	64%				1		
37	0.760	70%	1					
38	0.759	63%						1
39	0.758	63%					1	
40	0.757	64%		1				
41	0.751	63%				1		
42	0.744	65%				1		
43	0.731	62%	1					
44	0.731	63%		1				
45	0.723	60%			1			
46	0.715	66%					1	
47	0.692	61%		1				
48	0.689	56%					1	
49	0.688	57%						1
50	0.685	63%						1
51	0.670	54%				1		
52	0.670	55%					1	
53	0.663	55%		1				
54	0.655	54%		1				
55	0.599	45%		1				

56	0.578	45%			1			
57	0.572	44%		1				
58	0.547	42%						1
59	0.357	49%		1				

Table S3 - Top 3 research topics by theme and RPS ranks

Health systems' responsiveness to Migrant Needs	RPS Rank
1. Health systems’ ability to address needs of growing migrant populations in cities (including allocation of financial resources, medicines and staff)	5
2. Health systems’ response to internal migrants during COVID-19 crisis	20
3. Mental health burden and health systems response to (a) single male migrants in India’s metros (b) seasonal migrants and families (c) elderly migrants in urban areas (d) migrants working in unsafe/hazardous workspaces	23
Migrants' health and health access	
1. Availability, affordability and utilization of reproductive (incl maternal) and sexual health services in the public sector among: (a) internal migrant women (b) migrant adolescents	1
2. Healthcare access (including pathways, enablers/barriers) and outcomes among distinct migrant groups: a) seasonal (short-term) migrants b) long-term migrants (Settlers) c) Particularly Vulnerable Tribal Groups d) Nomadic ethnic communities (eg: Nats and Gujjars) (e) low-income migrants working in hazardous conditions (f) nomadic tribal populations	2
3. Uptake of existing health and social security schemes for migrant employees/families in the unorganised sector	3
Migration Health Discourse in Research	
1. The conceptual and theoretical underpinnings of research on migration and health: Exploring how policy, administrative and academic definitions of ‘migration’ and ‘migrants’ relates to on the ground	26
2. Challenges (and opportunities) in developing a research agenda on migration and health in India and influencing policy	31
3. Developing robust evidence base to understand the intersection of migration and health in the absence of datasets	33
Policies, Laws & Governance of Migration Health	
1. Gendered aspects of migration policies and impact on migrants’ health	4
2. Mapping/Evaluating central and state governments (sending & destination states) responses to the migrant crisis in the wake of COVID-19/ public health emergencies	8
3. Portability of nutrition & healthcare related entitlements (RSBY, ICDS, PDS) on crossing state borders	9
Social Determinants of Migrant Health	
1. Poor occupational health and impact on the reproductive health of migrant women in informal work sites	6
2. Gender differences in the prevalence of chronic disease risk factors and outcomes based on migration status (type and duration)	13
3. Work and living conditions (incl. sanitation) among low-income migrants and the resulting impact on their health (occupational health)	14

Tables S4 – Top 5 research topics per criterion prioritised by RPS

S6.1 Ranking by Criteria 1: Answerability: Addresses a significant gap in our knowledge of the field

Criteria Rank	Research Topic	Overall Rank
**1**	Availability, affordability and utilization of reproductive (incl maternal) and sexual health services in the public sector among: (a) internal migrant women (b) migrant adolescents	1
**2**	Healthcare access (including pathways, enablers/barriers) and outcomes among distinct migrant groups: a) seasonal (short-term) migrants b) long-term migrants c) Particularly Vulnerable Tribal Groups d) Nomadic ethnic communities (eg: Nats and Gujjars) (e) low-income migrants working in hazardous conditions (f) nomadic tribal populations	2
**3**	Gendered aspects of migration policies and impact on migrants' health	4
**4**	Mapping/Evaluating central and state government (sending & destination states) responses to the migrant crisis in the wake of COVID 19	8
**5**	Assessing nutrition status, access to mid-day meal schemes, and health outcomes of migrant children: (a) Accompanying parents who are seasonal migrants; b) left behind	7

S6.2 Ranking by Criteria 2: Effectiveness: Evidence can help improve migrants' health and/or reduce burden of ill-health/ disease

Criteria Rank	Research Topic	Overall Rank
**1**	Healthcare access (including pathways, enablers/barriers) and outcomes among distinct migrant groups: a) seasonal (short-term) migrants b) long-term migrants c) Particularly Vulnerable Tribal Groups d) Nomadic ethnic communities (eg: Nats and Gujjars) (e) low-income migrants working in hazardous conditions (f) nomadic tribal populations	2
**2**	Availability, affordability and utilization of reproductive (incl maternal) and sexual health services in the public sector among: (a) internal migrant women (b) migrant adolescents	1
**3**	Health systems ability to address needs of growing migrant populations in cities (including allocation of financial resources, medicines and staff)	5
**4**	Poor occupational health and impact on the reproductive health of migrant women in informal work sites	6

**5**	Assessing nutrition status, access to mid-day meal schemes, and health outcomes of migrant children: (a) Accompanying parents who are seasonal migrants; b) left behind	7

S6.3 Ranking by Criteria 3: Equity: has the potential to address inequities in processes and/or outcomes

Criteria Rank	Research Topic	Overall Rank
**1**	Healthcare access (including pathways, enablers/barriers) and outcomes among distinct migrant groups: a) seasonal (short-term) migrants b) long-term migrants c) Particularly Vulnerable Tribal Groups d) Nomadic ethnic communities (eg: Nats and Gujjars) (e) low-income migrants working in hazardous conditions (f) nomadic tribal populations	2
**2**	Availability, affordability and utilization of reproductive (incl maternal) and sexual health services in the public sector among: (a) internal migrant women (b) migrant adolescents	1
**3**	Gendered aspects of migration policies and impact on migrants' health	4
**4**	Uptake of existing health and social security schemes for migrant employees/families in the unorganised sector	3
**5**	Poor occupational health and impact on the reproductive health of migrant women in informal work sites	6

S6.4 Ranking by Criteria 4: Impact: has the potential to directly influence policy, programme/ practice & system-wide change

Criteria Rank	Research Topic	Overall Rank
**1**	Healthcare access (including pathways, enablers/barriers) and outcomes among distinct migrant groups: a) seasonal (short-term) migrants b) long-term migrants c) Particularly Vulnerable Tribal Groups d) Nomadic ethnic communities (eg: Nats and Gujjars) (e) low-income migrants working in hazardous conditions (f) nomadic tribal populations	2
**2**	Uptake of existing health and social security schemes for migrant employees/families in the unorganised sector	3
**3**	Availability, affordability and utilization of reproductive (incl maternal) and sexual health services in the public sector among: (a) internal migrant women (b) migrant adolescents	1
**4**	Health systems ability to address needs of growing migrant populations in cities (including allocation of financial resources, medicines and staff)	5
**5**	Gendered aspects of migration policies and impact on migrants' health	4

S6.5 Ranking by Criteria 5: Feasibility: Is feasible

Criteria Rank	Research Topic	Overall Rank
**1**	Availability, affordability and utilization of reproductive (incl maternal) and sexual health services in the public sector among: (a) internal migrant women (b) migrant adolescents	1
**2**	Uptake of existing health and social security schemes for migrant employees/families in the unorganised sector	3
**3**	Health systems ability to address needs of growing migrant populations in cities (including allocation of financial resources, medicines and staff)	5
**4**	Gendered aspects of migration policies and impact on migrants' health	4
**5**	Extent of inclusion of health and well-being of migrant workers in labour laws in India and extent of inclusion of migrants/migration in various health legislations and policies	10
